# Hebrew Self-Transcendence: Translation and Validation for Family Caregivers of People with Dementia

**DOI:** 10.1093/geroni/igaf122.3787

**Published:** 2025-12-31

**Authors:** Dalit Zaguri-Greener, Ruth Lopez, Nabel Gara, Lola Kolpin, Anna Zisberg

**Affiliations:** University of Haifa, Haifa, Hefa, Israel; MGH Institute of Health Professions, Sharon, Massachusetts, United States; Shoham Medical Center, Shoham Medical Center Pardes Hanna-Karkur, HaMerkaz, Israel; University of Haifa, Haifa, Hefa, Israel; University of Haifa, Haifa, Hefa, Israel

## Abstract

**Background:**

While family caregivers of individuals with advanced dementia often experience high levels of emotional and physical burden, less attention has been given to their sources of resilience and well-being. Tools like the Self-Transcendence Scale (STS) can help assess these positive dimensions, but culturally adapted versions are needed.

**Objective:**

To translate and culturally adapt the STS into Hebrew and evaluate its psychometric properties among family caregivers of nursing home residents with moderate-to-severe dementia.

**Methods:**

A sample of 175 Hebrew-speaking caregivers completed the STS. The scale underwent translation and cultural adaptation, followed by exploratory and confirmatory factor analyses.

**Results:**

Factor analyses revealed a four-factor structure: Intrapersonal, Interpersonal, Transpersonal, and Temporal, aligned with Reed’s theoretical framework. Convergent validity was demonstrated, with higher ST scores significantly associated with lower caregiver burden (r = –.34, p < .001). Internal consistency was acceptable (Cronbach’s alpha = .72). The Temporal dimension emerged as a key contributor, reflecting caregivers’ integration of past, present, and future experiences.

**Conclusions:**

The Hebrew STS is a valid and reliable tool for assessing self-transcendence among family caregivers. It offers new insights into their psychological adaptation and may support future cross-cultural research and intervention development.

